# Selective vulnerability of von Economo neurons in frontotemporal dementia

**Published:** 2008

**Authors:** Ricardo Nitrini

In many types of neurodegenerative disease there is selective vulnerability of groups of neurons that show evidence of degenerative change long before other neighboring neurons. In Alzheimer’s disease, neurons in layer II of the transentorhinal and entorhinal cortex show neurofibrillary tangles while neurons in the other layers of the same region remain normal. In Parkinson’s disease, selected populations of neurons in the medulla oblongata, pontine tegmentum, olfactory bulb, anterior olfactory nucleus and pars compacta of the substantia nigra are involved earlier in the degenerative process than other neurons in adjacent areas. Until recently, the most vulnerable neurons in frontotemporal dementia (FTD) were not known. Studies conducted by Seeley and colleagues headed by Bruce L. Miller, from the Memory and Aging Center of the University of California at San Francisco, have found very interesting results on this matter.^1-3^

Layer Vb of the cingulate cortex and orbito frontoinsular cortex of the adult brain contain large bipolar spindle-shaped neurons not present in other brain areas. These neurons were described by von Economo and Koskinas in 1925 (cit. by Viskontas et al., 2007)^2^ as spindle cells and, although they have long axons, their connections are not yet known. There are several fascinating observations related to these neurons, now called von Economo neurons (VENs). For instance, they are present only in man and other mammals with large brains such as great apes and a few species of whales; VENs are larger and more numerous in man than in the great apes, and their number and size decreases according to the distance from man in the evolutionary chain; VENs appear later in development, increasing in size and number from birth until the age of four to eight years, being more numerous in the right hemisphere.^1-3^

VENs are precociously involved in FTD and may be the most vulnerable neuron to the degenerative processes that cause this complex behavioral syndrome.^1^ The function of VENs are as yet unknown but the finding that VENs express dopamine (D3), serotonin (1b/2b) and vasopressin receptors is of great interest because the neurotransmitters involved with these receptors are related to behaviors commonly disturbed in FTD^3^. This represents a new field of research with high potential for significant findings in the near future.

**Ricardo Nitrini**
